# Factors associated with return of home oral fluid kits by suspected cases of measles, cohort study, London and South East of England, 2016–2018

**DOI:** 10.1017/S095026882300095X

**Published:** 2023-06-23

**Authors:** Iina Hiironen, Antoaneta Bukasa, Anand Fernandes, Katie Allen, Claire Winslade, Anita Bell, Ellie Maslen, Yimmy Chow, Jonathan Fok, Sarah Lock, Rachel Mearkle, Amanda Wright, Neville Q. Verlander, Sooria Balasegaram, Maria Saavedra-Campos

**Affiliations:** 1 Field Service, South East and London, UKHSA, London, UK; 2 UKHSA National Immunisation, Hepatitis and Blood Safety Department, National Infection Service, UKHSA, London, UK; 3 Hampshire and Isle of Wight Health Protection Team, UKHSA, Southampton, UK; 4 Kent and Medway Health Protection Team, UKHSA, Kent, UK; 5 North East and Central London Health Protection Team, UKHSA, London, UK; 6 North West London Health Protection Team, UKHSA, London, UK; 7 Surrey and Sussex Health Protection Team, UKHSA, Surrey, UK; 8 Thames Valley Health Protection Team, UKHSA, Oxford, UK; 9 Statistics, Modelling and Economics Department, National Infection Service, UKHSA, UK

**Keywords:** control, elimination, measles (rubeola), oral fluid kit

## Abstract

A testing rate for measles above 80% is required by the WHO European Region Measles Elimination strategy to verify elimination. To comply with this rate, we explored factors associated with the return of oral fluid kits (OFK) by suspected measles cases. We described the cases and conducted a mixed-effects analysis to assess the relationship between socio-demographic and public health management characteristics and the likelihood of returning an OFK to the reference laboratory. Of 3,929 cases who were sent a postal OFK, 2,513 (67%) returned the kit. Adjusting for confounding, registration with a general practitioner (GP) (aOR:1.48, 95%CI:1.23–1.76) and living in a less deprived area (aOR:1.35, 95%CI:1.04–1.74) were associated with an increased likelihood of returning the OFK. The odds of returning the OFK also increased if the HPT contacted the parents/guardians of all cases prior to sending the kit and confirmed their address (aOR:2.01, 95%CI:1.17–3.42). Cases notified by a hospital (aOR:1.94, 95%CI:1.31–2.87) or GP (aOR:1.52; 95%CI:1.06–2.16) also had higher odds of returning the OFK. HPTs may want to consider these factors when managing suspected cases of measles since this may help in increasing the testing rates to the WHO-recommended level.

## Introduction

In June 2017, based on data from 2014–16, the World Health Organization (WHO) declared that the United Kingdom (UK) had achieved measles elimination status for the first time, defined by WHO as the absence of circulating measles for at least 12 months in the presence of high vaccine coverage and an effective surveillance system [1]. However, a marked increase in confirmed measles cases in 2018 and declining measles-mumps-rubella vaccine (MMR) coverage led to the re-establishment of indigenous measles transmission and the loss of the UK’s measles elimination status in September 2019. Annual coverage data for 2018–19 showed that uptake of the MMR1 vaccine in England at 5 years stood at 94.5%, below the 95% WHO-recommended threshold for herd immunity, particularly important to protect vulnerable groups unable to be vaccinated themselves, with some local authorities in London (12/32) recording uptake below 90% [2].

To monitor case numbers, transmission routes, and potential importation, it is essential that every suspected measles case is sent an oral fluid kit (OFK) by their local health protection team (HPT) upon being notified as a case. The OFK is carried out by the case, their parent/guardian, or a healthcare professional and posted to the UK Health Security Agency (UKHSA) reference laboratory, where it is tested for anti-measles IgM, measles IgG, and/or measles RNA [3]. OFKs and IgM serology testing are regarded by WHO as the only two tests acceptable for confirming or discarding suspected measles cases. A testing rate of at least 80% of suspected cases is one of the indicators required by WHO as a measurement of a well-performing measles surveillance system [4]. In 2018, an audit of OFK practice by HPTs found that, while a consistently high proportion of kits (96%) was sent to suspected measles cases with little variation across HPTs, the proportion received and tested by the reference laboratory was much lower and varied considerably by HPT, with only 2 out of 16 HPTs exceeding 80% [5]. There was also local variability in processing OFKs within HPTs, with the audit calling for OFKs to be sent promptly regardless of diagnostic certainty or the results of local laboratory testing, as well as calling for a review of the standard letter accompanying the kit.

A search of the literature uncovered no information exploring ways to maximise the rate of completed OFKs for measles returned by post to the reference laboratory. The aim of this study is to identify factors influencing the return of completed OFKs by recipients to the reference laboratory in London and the South East of England, which is key to achieving the > = 80% WHO testing target, with a view to formulating recommendations to improve the number of OFK returns.

## Methods

### Data sources, study design, and study period

Using HPZone™ (inFact UK Ltd, Shipley, Yorkshire), the case management system employed by UKHSA HPTs, we conducted a retrospective cohort study, extracting all cases of measles notified to the relevant HPTs between 1 January 2016 and 31 December 2018 in London and the South East of England.

For the creation of the outcome variables, we used data from the reference laboratory, including the results of the OFK sent for testing.

We also used data on the processes and practices associated with the use of OFKs for suspected measles cases from an audit administered to HPTs in 2018, which contained detailed information on the different actions taken by each team (Table 1).

**Table 1.** Processes and practices of different HPTs in dealing with OFKs, depending on the confidence of the initial case report (i.e., confirmed, probable, or possible) and according to the audit on processes and practices associated with oral fluid testing and suspected cases of measles led by the Measles and Rubella Elimination Health Protection Teams and Field Epidemiology Service Working Group on behalf of the Vaccine Preventable Diseases Health Protection Team Leads Group, July 2018

South East of England				
Health Protection Team	A	B	C	D
OFK sent^a^	Yes (all)	Yes (all)	Always (possible, probable) Sometimes (confirmed)	Yes (all, excluding local urgent tests)
Contact prior	Yes (probable, confirmed)	Yes (all)	Yes (probable, confirmed), No (possible)	Yes (probable, confirmed)
	No (possible)			No (possible)
Address check	No (all)	Yes (all)	Yes (probable, confirmed)	Yes
Follow up^d^	Yes (probable, confirmed)	No (all)	Yes (probable, confirmed)	Yes (probable only)
Prior information: additional information provided to cases or their parents/guardians by the HPT before sending out an oral fluid kit				
Purpose of the kit	Yes (probable, confirmed)	Yes (all)	Yes (probable only)	Yes (probable only)
How to take the test	Yes (probable, confirmed)	Yes (all)	Yes (probable only)	Yes (probable only)
How to fill the form	Yes (probable, confirmed)	No (all)	Yes (probable only)	No
Advantages	Yes (probable, confirmed)	Yes (all)	Yes (probable only)	Yes (probable only)
Public health importance	Yes (probable, confirmed)	No (all)	Yes (probable only)	Yes (probable only)
How they will receive the form	Yes (probable, confirmed)	Yes (all)	Yes (probable only)	Yes (probable only)
London				
Health Protection Team	E	F	G	
OFK sent^a^	Yes (all)	Yes (all)	Yes (all)	
Contact prior^b^	Yes (all)	Yes (all)	Yes (all)	
Address check^c^	No (all)	No (all)	No (all)	
Follow up^d^	No (all)	No (all)	No (all)	
Prior information: additional information provided by the HPT to cases or their parents/guardians before sending out an oral fluid kit				
Purpose of the kit	Yes (all)	Yes (all)	Yes (all)	
How to take the test	Yes (all)	Yes (all)	Yes (all)	
How to fill the form	Yes (all)	No (all)	No (all)	
Advantages	Yes (all)	No (all)	No (all)	
Public health importance	Yes (all)	No (all)	No (all)	
How they will receive the test	Yes (all)	Yes (all)	Yes (all)	

a Oral fluid kit

b Verbally contact a case or their parent/guardian to inform them that an oral fluid testing kit is going to be sent to them

c Confirm the address where the kit is to be sent with the recipient.

d Schedule a follow-up action to monitor completion of an OFK.

We obtained data on rural–urban classification and the Index of Multiple Deprivation (IMD), which ranks Lower-Layer Super Output Areas (LSOA) in England from the most to the least deprived by postcode from the Office of National Statistics [6]. We allocated an IMD score and rural–urban classification to each case based on their postcode.

### Study population

We included in our study population any cases reported to the HPTs within the study area where a clinician suspected measles as a diagnosis, regardless of the classification of the case at the time of reporting, that is, possible, probable (more clinical or epidemiological certainty), confirmed (tested by a local laboratory as opposed to tested by the reference laboratory), or discarded (negative result reported by the reference laboratory).

### Exclusion criteria

We excluded any cases where the Local Authority (LA) of residence was not within the study area (South East of England and London), cases that were reported with an initial diagnosis other than measles, and cases who had not been sent an OFK.

### Data linkage and outcome variable

We linked the cases’ HPZone data to the results from the reference laboratory in order to create the binary outcome variable reflecting whether or not the OFK was returned.

### Statistical analysis

We performed a descriptive analysis of all the explanatory variables to explore any gaps. For those variables with missing data, we statistically tested whether or not the data was missing completely at random by creating a binary variable for ‘missing’ for each one of these variables. Then we tested whether or not ‘missing’ was associated with the outcome and whether an analysis of complete cases was adequate for our data to minimise any potential biases introduced by the missing data.

We performed a single and multivariable multilevel logistic regression analysis with HPT as the upper level, where the association between each explanatory variable and the outcome was examined one at a time without adjustment for any other explanatory variable in the former analysis. Any variable from this analysis with p-value < 0.2 in addition to age was then brought into the multivariable analysis. We used a backwards approach to identify a final model, eliminating variables with the highest Chi-squared values first and examining for possible confounders at each step, starting with the explanatory variables with minimal missing data. Next, we added the variables with missing data one by one (notified by and hospitalised at the time of reporting) and repeated the process above. This last part of the analysis resulted in a large reduction of observations in the model; hence, more than one final model is presented. The above multivariable analysis was repeated by assuming independence as the variance component was close to zero and the two sets of results were compared. Since there was little difference between the two, the results of the independence analysis have been presented. In both the single and the multivariable analysis, the appropriate functional form for the association between the continuous variable of age and the outcome on the logit scale was ascertained by beginning with a cubic function and successively simplifying to the next simplest function not fitting significantly worse, with these steps being performed prior to variable removal for the latter analysis. Statistical significance was determined by means of the likelihood ratio test (LRT), with the significance level taken to be 5%.

The analysis was performed using STATA 15 (StataCorp. 2015. Stata Statistical Software: Release 14. College Station, TX: StataCorp LP).

## Results

### Missing data

We identified several variables with high levels of missing data (Table 2), including whether or not a case was hospitalised (60% missing) at the time of notification, whether or not the case was up to date with MMR vaccination (44% missing), and who notified the case (46% missing). The variable representing whether a case’s residence was in an urban or rural area also contained missing data but less than the variables above (5% missing). The results exploring the relationship between data gaps and the outcome showed that missing data was associated with the outcome, and therefore our data is not missing completely at random. Hence, we can only assume that our data is, at best, missing at random. We did not perform imputation as the fraction of missing information in our data would have required a bigger number of variables to be able to provide reliable imputations.

**Table 2.** Descriptive and single variable analysis of explanatory variables potentially associated with the outcome of having received an oral fluid test by the reference laboratory, London and the South East of England, 2016 to 2018 (*n* = 3,929)

		Number of individuals who returned the OFK^a^ (*n* = 2,513)			Number of individuals who did not return the OFK^a^ (*n* = 1,416)					
Variable		Total	Exposed	%	Total	Exposed	%	cOR^b^	95% C.I.^c^	LRT^d^
		Range (1–85)			Range (1–65)					
		Inter-quartile range (1–15)			Inter-quartile range (1–56)					
Age (years)		Median 3	Mean 10		median 4	Mean 10		0.99	[0.99–1.00]	0.33
Gender										
	Female	2,513	1,121	44.61	1,416	668	47.18	0.89	[0.78–1.01]	0.09
	Missing data	0			0					
Urban										
	Urban	2,513	2,253	89.65	1,416	1,266	89.41	0.83	[0.60–1.14]	0.2
	Missing data	2,513	105	4	1,416	86	6			
Hospitalised										
	Yes	2,513	175	6.96	1,416	101	7.13	0.9	[0.68–1.18]	0.5
	Missing data	2,513	1,478	59	1,416	867	61			
Travel										
	Yes	2,513	231	9.19	1,416	110	7.77	1.1	[0.85–1.43]	0.439
	Missing data	2,513	1,352	55	1,416	817	58			
Vaccinated^e^										
	Vaccinated for age	2,065	637	30.85	1,179	306	25.95	1.11	[0.92–1.35]	0.262
	Missing data	2,065	860	42	1,179	568	48			
Registered with a GP (GP details entered on HPZone)										
	Yes	2,513	2,093	83.29	1,416	1,066	75.28	1.59	[1.35–1.87]	<0.001
	Missing data	2,513	0		1,416	0				
Locally tested										
	Yes	2,513	1	0.04	1,416	172	12.15	0.003	[0.00–0.02]	<0.001
	Missing data	2,513	0		1,416	0				
Notified by										
	Other^f^	2,513	109	4	1,416	68	5	Ref		0.027
	Hospital or laboratory	2,513	361	14	1,416	173	12	1.30	[0.91–1.85]	
	Formal notification^h^	2,513	270	11	1,416	156	11	1.08	[0.75–1.54]	
	GP	2,513	691	28	1,416	294	21	1.47	[1.05–2.04]	
	Missing data	2,513	1,082	43	1,416	725	51			
Deprivation quintile										
	1	2,489	432	17	1,416	266	19	Ref		0.1
	2	2,489	749	30	1,416	477	34	0.96	[0.80–1.17]	
	3	2,489	485	19	1,416	262	19	1.10	[0.88–1.37]	
	4	2,489	410	16	1,416	205	14	1.16	[0.92–1.47]	
	Least deprived	2,489	413	17	1,416	187	13	1.28	[1.00–1.62]	
	Missing data	2,489	24	1	1,416	19	1			
Public health action: processes and practices associated with oral fluid testing and suspected cases of measles depending on the initial diagnosis (possible, probable, or confirmed)										
Contact prior (probable and confirmed cases), address check (all cases), follow up (probable)		2,513	73	3	1,416	41	3	Ref		0.06
Contact prior only (all cases)		2,513	1,619	64	1,416	984	69	0.92	[0.60–1.42]	
Contact prior and address check (all cases)		2,513	141	6	1,416	45	3	1.76	[1.01–3.06]	
Contact prior, address check, and follow up (probable and confirmed cases)		2,513	534	21	1,416	273	19	1.09	[0.68–1.74]	
Contact prior, follow up (probable and confirmed cases)		2,513	146	6	1,416	73	6	1.12	[0.66–1.89]	
Missing data		0	0	0	1,416	0				
Information prior^g^: additional information provided by the HPT to cases or their parents/guardians before sending out an oral fluid kit depending on their assessment of the confidence of the case at the time of reporting (possible, probable, or confirmed), including six levels of information; the purpose of the kit (pk), how to take the test (take), how to complete the form included in the test for sending back to the reference laboratory (fill), the advantages of taking the test (adv), the public health importance of the test (ph), and how they will receive the result (get).										
All information to all cases		2,513	605	24	1,416	433	31	0.78	[0.52–1.17]	<0.001
All information only to probable and confirmed cases		2,513	146	6	1,416	73	5	1.12	[0.69–1.80]	
All information to all probable cases		2,513	534	21	1,416	273	19	1.10	[0.72–1.65]	
pk-take-get to all cases		2,513	1,014	40	1,416	551	39	1.03	[0.69–1.53]	
pk-take-adv-ph-get to all probable cases		2,513	73	3	1,416	41	3	Ref		
pk-take-adv-get to all cases		2,513	141	6	1,416	45	3	1.76	[1.05–2.92]	
Missing data		2,513	0	0	1,416	0	0	0		

a Oral fluid kit

b Crude odds ratio.

c Confidence interval.

d Likelihood ratio test.

e Numbers do not include cases under one year of age.

f School, nursery, care home, emergency services, environmental health, member of the public, public health services, self-reported, and workplace.

g Prior information: additional information the HPT provides to cases or their parents/guardians before sending out an oral fluid kit

h Enter on HPZone as a formal notification.

### Multilevel analysis

We looked at potential clustering at the health protection team level and found small contributions to the estimates that could be explained by the clustering variable. We ran a single variable analysis and multivariable analysis both with and without accounting for clustering and found very similar estimates. We therefore conducted an independence multivariable analysis and presented the estimates obtained by this method.

### Single variable analysis

A total of 3,929 cases were left in the study after the exclusion of the 24 cases as outlined in the methods. For 2,513 (67%) of the 3,929 cases, an OFK was received by the reference laboratory. The cases for whom the OFK was returned were on average of a similar age to those for whom it was not returned (median 3 years of age compared with 4 years) and had a 64% increase in the odds to be registered with a general practitioner (GP) (Odds Ratio (OR) 1.64, 95% Confidence Interval (CI) 1.39 to 1.93). Cases residing in urban areas showed 27% lower odds of returning the OFK compared with cases living in rural areas (OR 0.73, 95%CI 0.54, 0.99). The odds of the OFK being returned decreased (OR 0.003, 95%CI 0.0004–0.02) if the case had been tested in a local laboratory at the time of being notified compared with cases who were not tested at the time of being reported. However, this result is based on only one case. Cases notified by a GP had a 47% increase in the odds of returning the OFK (OR 1.47, 95% CI 1.05, 2.04) compared to cases notified by other reporters (school, nursery, care home, emergency services, environmental health, member of the public, public health services, self-reported, and workplace), excluding hospitals and laboratories or the ones recorded as formal notifications on HPZone. Cases living in postcodes associated with least deprived areas had a 36% increase in the odds of the OFK being returned (OR 1.36, 95%CI 1.07, 1.72) compared to cases living in postcodes associated with more deprived areas.

Regarding the public health management of the measles cases and the processes and practices associated with the use of OFK, the odds of the OFK being returned to the reference laboratory were higher (OR 1.76, 95%CI, 1.05, 2.92) in the areas where HPTs routinely

- contacted all the cases or parent/guardian beforehand to let them know that the OFK was in the post,
- and confirmed the address of all the cases, regardless of whether they were suspected (possible and probable) or locally confirmed at the time of reporting.

This was in contrast to the areas where HPTs checked the address for all the cases, but only contacted locally confirmed and probable cases beforehand, and only set up a follow-up action to monitor completion of an OFK test for probable cases.

Regarding the information provided to the case when contacted by the public health teams, the odds of the OFK being returned were higher in the areas where information on

- the purpose of the test,
- how to take the test,
- the advantages of taking the test, and
- how cases will receive the test

was provided to all the cases irrespective of whether they were suspected (possible or probable) or locally confirmed at the time of notification. This is in contrast to areas where all the information above, together with the public health importance of taking the test, was discussed only with cases originally assessed as probable at the time of notification (OR 1.76, 95%CI, 1.05, 2.97).

### Multivariable analysis

We presented the results of two models, excluding (Table 3) or including (Table 4) variables with missing data.

**Table 3.** Multivariable analysis including only explanatory variables with limited missing data, London and the South East of England, 2016 to 2018 (*n* = 3,727)

Variable	aOR^a^	95% C.I.^b^	LRT^c^
Age	1.00	[0.99-1.01]	0.16
Gender	0.89	[0.77–1.02]	0.11
Registered with a GP	1.48	[1.23–1.76]	<0.001
Urban	0.89	[0.63–1.24]	0.49
Locally tested	0.00	[0.0004–0.021]	<0.001
Deprivation quintile			
1	Ref		
2	1.00	[0.81–1.23]	0.06
3	1.19	[0.94–1.49]	
4	1.21	[0.94–1.55]	
Least deprived	1.35	[1.04–1.74]	
Public health action: processes and practices associated with oral fluid testing and suspected cases of measles depending on the initial diagnosis (possible, probable, or confirmed)			
Contact prior (probable and confirmed cases), address check (all cases), follow up (probable cases)	Ref		0.07
Contact prior only (all cases)	1.24	[0.82–1.87]	
Contact prior and address check (all cases)	2.01	[1.17–3.42]	
Contact prior, address check, and follow up (probable and confirmed cases)	1.28	[0.83–1.95]	
Contact prior (probable and confirmed cases), address check (all cases), follow up (probable cases)	1.37	[0.82–2.25]	

a Adjusted odds ratio.

b Confidence interval.

c Likelihood ratio test.

**Table 4.** Multivariable analysis including explanatory variables with limited missing data and explanatory variables with missing data, London and the South East of England, 2016 to 2018 (*n* = 2,023)

Variable	aOR^a^	95% C.I.^b^	LRT^c^
Age	1.01	[0.998-1.014]	0.095
Gender	0.69	[0.564–0.847]	<0.001
Registered with a General Practitioner	1.44	[1.116–1.845]	0.005
Urban	0.74	[0.455–1.201]	0.21
Locally tested	0.00	[0–.]	
Deprivation quintile			
1	Ref		0.109
2	1.05	[0.782–1.420]	
3	1.19	[0.852–1.653]	
4	1.35	[0.932–1.961]	
Least deprived	1.55	[1.069–2.232]	
Public health action: processes and practices associated with oral fluid testing and suspected cases of measles depending on the initial diagnosis (possible, probable, or confirmed)			
Contact prior (probable and confirmed cases), address check (all cases), follow up (probable cases)	Ref		0.019
Contact prior only (all cases)	1.67	[0.929–3.012]	
Contact prior and address check (all cases)	2.55	[1.268–5.145]	
Contact prior, address check, and follow up (probable and confirmed cases)	1.25	[0.673–2.305]	
Contact prior (probable and confirmed cases), address check (all cases), follow up (probable cases)	1.32	[0.661–2.651]	
Notified by			
Other^d^	Ref		<0.001
Hospital or laboratory	1.94	[1.311–2.871]	
Formal notification^e^	1.01	[0.679–1.493]	
General Practitioner	1.52	[1.060–2.166]	

a Adjusted odds ratio.

b Confidence interval.

c Likelihood ratio test.

d School, nursery, care home, emergency services, environmental health, member of the public, public health services, self-reported, and workplace.

e Enter on HPZone as a formal notification.

#### First model with more observations, excluding variables with missing data

In the more complete model (*n* = 3,727), and after adjusting for confounding, being registered with a GP remained significantly associated with returning the OFK. However, just over the significance threshold, cases living in the least deprived areas showed higher odds of returning the OFK (Table 3). Although not all the levels appeared to be significant, the odds of returning the OFK showed an increase as the level of deprivation decreased (Table 3). Similarly, the areas where all the cases, irrespective of their initial classification (possible, probable, or confirmed locally), were contacted prior to the OFK being sent and had their address checked had higher odds of returning the OFK (Table 3). We kept the variable urban in the model, even though it was not significant. We also ran a model without it and the estimates were not substantially different, so we decided to present the model including the variable *urban.*

#### Second model with fewer observations, including the variables with missing data

A total of 2,023 observations were included in this model. The odds of returning the OFK were increased if the case was registered with a GP, or if the case lived in an area where all the cases, irrespective of their initial classification (possible, probable, or confirmed locally) were contacted prior to being sent an OFK and had their address checked. Likewise, cases that were notified by a hospital or laboratory or a GP had higher odds of returning the OFK (Table 4). In this model, being female and living in an urban area were associated with lower odds of returning the OFK (Table 4). Although not significant, the odds of returning the OFK remained increased for cases in the least deprived areas, with the odds increasing as the deprivation decreased (Table 4).

Since the independent variable *notified by* was significant in the second model, we also ran the first model restricting it to only the observations where the variable *notified by* was not missing and the estimates obtained were not substantially different.

## Discussion

Our findings show that cases of suspected measles registered with a GP and/or living in less deprived areas showed higher odds of returning the OFK to the reference laboratory. Regarding the local processes in response to measles cases, OFKs had higher odds of being returned to the reference laboratory in those areas where cases were contacted prior to sending the OFK or where cases were contacted and had their address confirmed.

In our second model including the variables with missing data, therefore, based on fewer observations, in addition to being registered with a GP, cases notified by a hospital/laboratory or GP had higher odds of returning the OFK. Regarding the processes related to the OFK, the areas that routinely contacted cases prior to sending the OFK and areas where cases were contacted and had their address checked showed higher odds of returning the OFK to the reference laboratory.

Although there is extensive literature on the use of OFK and rapid diagnostic tests for measles surveillance, we could not find any previous research identifying factors that could predict whether an OFK would be returned by a suspected measles case or case with any other infection, such as rubella and mumps, for which an OFK is routinely sent out in the post [7–9]. However, it seems plausible that the factors discussed above might genuinely be associated with returning the OFK to the reference laboratory. Efforts to increase the number of OFKs returned might be better directed to cases who are not known to be registered with a GP or who live in more deprived areas. Additionally, cases who are not initially reported to public health by a hospital, laboratory, or GP may also benefit from a follow-up action. However, this finding was based on fewer observations.

### Limitations

The main limitation of our study is the extent to which data is missing for some of our variables. We were able to ascertain that our data was not missing completely at random, and so, as a result, we performed a complete cases analysis only. However, due to the missing data, we cannot exclude that our results might be biased. Regarding the variable ‘hospitalised’, we believe the impact on our results of missing data in this field is likely to be small as measles cases are seldom hospitalised. We also believe that the impact on our results of missing data in the field relating to who notified the case would remain small as measles cases are most commonly reported by a GP.

It is possible that some samples received in the reference laboratory may have been sent by a hospital or an A&E department, overestimating the number of cases where the OFK was returned and potentially biasing our results. However, we believe the impact on our results to be small since hospitalisation of measles cases is uncommon and suspected measles cases tend to be diagnosed in primary care.

Additionally, where an exposure to a vulnerable contact has been identified and an urgent result needed, the HPT will send an OFK via courier to the case or parent/guardian and then onto the reference laboratory. This may overestimate our results, although we believe the impact is likely to be small.

Cases who had a sample taken during admission to hospital or after attending A&E might have a higher odds of returning an OFK to the reference laboratory if sent one, which was normal practice. As above, we anticipate this to be a small number of cases with not much impact on our estimates.

Similarly, there could be other factors associated with the outcome which are not part of our study. Anecdotally we know that some of the OFK used at the time of the study might have been too big to go through some letter boxes, meaning that they may have never reached the case in the first place. Also, the time from when the test was sent to when the case or parent/guardian received it may have had an impact on the likelihood of returning it. This could not be explored as we did not have the data.

Although the results suggest that cases that are called and had their address checked had higher odds of returning the OFK, it is highly likely that these calls include other public health advice that may also have an impact on whether a kit is returned or not. It is highly plausible that it is a combination of both actions that has an impact, as opposed to the address confirmation only.

## Conclusions

Laboratory investigation of all suspected cases of measles, together with clinical, laboratory, and epidemiological data, is essential for final case classification and investigation of chains of transmission on the road to elimination. Our study suggests that efforts to increase the number of OFKs that are returned to the reference laboratory could target suspected cases of measles who are not registered with a GP and live in more deprived areas. In terms of the processes associated with sending the OFK, verbally contacting a case or their parent/guardian to inform them that an oral OFK is going to be sent and verifying the address in advance is likely to have good results. Although not explored in our study, a follow-up action several days after the OFK is sent in the post to contact the case or parent/guardian if the case is not registered with a GP or lives in more deprived areas, may improve the proportion of OFKs returned, and this might be achieved within existing resources.

## Data Availability

No data are available. Data cannot be made publicly available for ethical and legal reasons, that is, public availability would compromise patient confidentiality, directly or via deductive disclosure.

## Supplementary material

For supplementary material accompanying this paper visit http://doi.org/10.1017/S095026882300095X. click here to view supplementary material

## Confidentiality

All documents and files containing patient-identifiable information will be handled and stored in compliance with the Data Protection Act (2018) and guidelines established by the local Caldicott guardian.
